# 3-Acetyloxy-2-cyano-2-(alkylaminocarbamoyl)propyl Groups as Biodegradable Protecting Groups of Nucleoside 5´-mono-Phosphates

**DOI:** 10.3390/molecules16010552

**Published:** 2011-01-14

**Authors:** Mikko Ora, Anne Mäntyvaara, Harri Lönnberg

**Affiliations:** Department of Chemistry, University of Turku, FIN-20014 Turku, Finland

**Keywords:** prodrug, nucleotide, biodegradable, protecting group

## Abstract

Thymidine 5´-bis[3-acetyloxy-2-cyano-2-(2-phenylethylcarbamoyl)propyl]phosphate (**1**) has been prepared and the removal of phosphate protecting groups by hog liver carboxyesterase (HLE) at pH 7.5 and 37 °C has been followed by HPLC. The first detectable intermediates are the (*R*_P_)- and (*S*_P_)-diastereomers of the monodeacetylated triester **14**, which subsequently undergo concurrent retro-aldol condensation to diester **4** and enzyme-catalyzed hydrolysis to the fully deacetylated triester **15**. The former pathway predominates, representing 90% of the overall breakdown of **14**. The diester **4** undergoes the enzymatic deacetylation 700 times less readily than the triester, but gives finally thymidine 5´-monophosphate as the desired main product. To elucidate the potential toxicity of the electrophilic 2-cyano-*N*-(2-phenylethyl)acrylamideby-product **17** released upon the deprotection, the hydrolysis of **1** has also been studied in the presence of glutathione (GSH).

## 1. Introduction

**Scheme 1:**
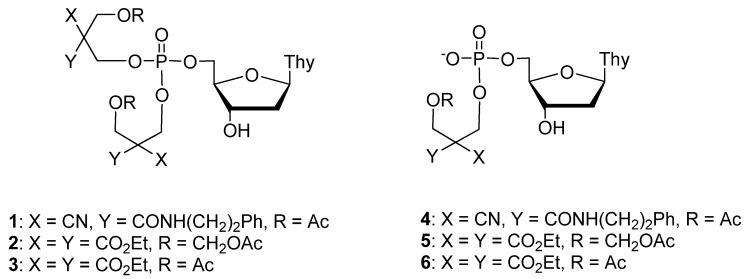
Structures of compounds **1**-**6**.

Masking of the ionic phosphate moiety of nucleotide analogues with enzyme-labile protecting groups offers a viable prodrug approach [1,2,3,4,5,6]. Enzymatic deacylation of one of the masking groups of a nucleoside 5´-phosphotriester triggers a concomitant chemical cleavage of the remnants of the protecting group, yielding nucleoside 5´-phosphodiester [7,8,9,10,11,12,13,14,15,16,17,18,19,20,21,22,23]. The negatively charged diester undergoes enzymatic deacylation much less readily than the parent triester [24,25,26,27,28], and hence, this latter step constitutes a bottleneck for the release of the 5´-monophosphate. Our previous results with nucleoside 5´-phosphotriesters **2** and **3** and the respective diesters **5 **and **6** have revealed that even a rather small structural modification, viz. introduction of an additional –CH_2_O- group between the enzyme labile ester function and the 2,2-diethoxycarbonyl substituted propyl group, accelerates the enzymatic deacetylation of both the triester and diester [29]. When using hog liver carboxyesterase, the acceleration is 40-fold. In fact, the deacetylation of diester **5** is already so rapid that the subsequent chemical step, *viz*. departure of 3-hydroxy-2,2-bis(ethoxycarbonyl)propyl group by retro-aldol condensation (Scheme 1), becomes rate-limiting. The rate of this step may, however, be tuned within wide limits by the polar nature of the 2-substituents [30,31,32,33]. For example, 2-cyano-2-carbamoyl disubstitution markedly accelerates the retro-aldol condensation compared to bis(ethoxycarbonyl) substitution. The primary aim of the present study is to find out whether this combination of 2-substituents also allows so facile enzymatic deacetylation of the diester that, taken together with rapid chemical retro-aldol condensation, an efficient pro-drug strategy is obtained. For this purpose, thymidine 5´-bis[3-acetyloxy-2-cyano-2-(2-phenylethylcarbamoyl)propyl]phosphate (**1**) has been prepared as a model compound and its hydrolysis initiated by hog liver carboxyesterase (HLE) has been followed by HPLC.

The potentially toxic by-products, such as formaldehyde and electrophilic alkylating agents, released upon breakdown of the biodegradable protecting groups form a general problem of pro-drug strategies. These by-products are believed to be captured by glutathione (GSH) present in cells at a high concentration. For example, aryl vinyl ketones released from HepDirectprodrugs in hepatocytes have been shown to undergo rapid conjugation with GSH in the mice serum [34]. Glutathione has also been proposed to react with formaldehyde in human cells [35]. *S*-Hydroxymethylglutathione formed is oxidized by formaldehyde dehydrogenase to *S*-formylglutathione which is further hydrolyzed to formate by *S*-formylglutathione hydrolase, regenerating free GSH [36]. The secondary aim of the present study is to evaluate the alkyating ability of 2-cyano-*N*-(2-phenylethyl)acrylamide (**17**) formed upon the deprotection of **1**. For this reason, the HLE-triggered deprotection in the presence of GSH has been followed by HPLC-ESI-MS.

## 2. Results and Discussion

### 2.1. Synthesis

**Scheme 2:**
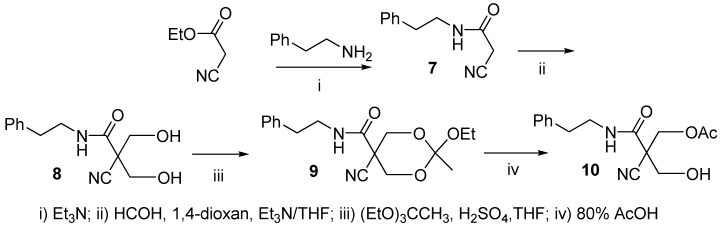
Preparation of 3-acetyloxy-2-cyano-2-hydroxymethyl-*N*-(2-phenylethyl)propanamide (**10**).

**Scheme 3:**
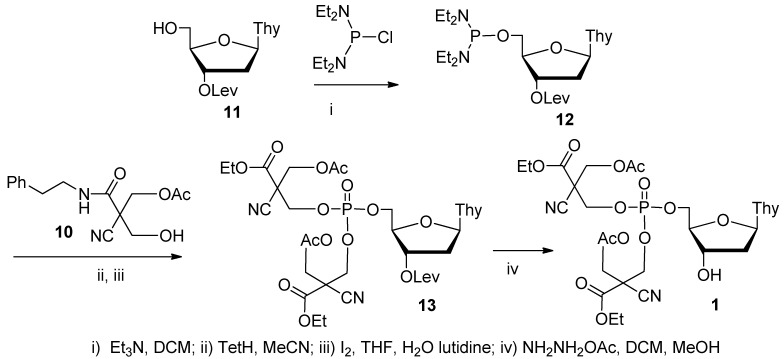
Preparation of thymidine 5´-bis[3-acetyloxy-2-cyano-2-(2-phenylethyl-carbamoyl)propyl]phosphate (**1**).

2-cyano**-***N*-(2-phenylethyl)acetamide (**7**), prepared from ethyl cyanoacetate by acyl substitution with (2-phenylethyl)amine, was bis(hydroxymethylated) by a procedure described earlier [37]. The 2-cyano-3-hydroxy-2-(hydroxymethyl)-*N*-(2-phenylethyl)propanamide (**8**) thus obtained was then converted to orthoacetate **9** and finally hydrolyzed to 3-acetyloxy-2-cyano-2-hydroxymethyl-*N*-(2-phenylethyl)propanamide (**10**), essentially as described earlier (Scheme 2) [38]. Alcohol **10** was isolated and used in tetrazole promoted alcoholysis of 3´-*O*-levulinoylthymidine 5´-(*N*,*N*-diethylaminophosphoramidite (**12**) [29] (Scheme 3). The phosphite ester obtained was oxidized with iodine in a mixture of THF, water and lutidine to the corresponding phosphate ester **13** and the levulinoyl protecting group was removed with hydrazinium acetate in a mixture of dichloromethane and MeOH.

### 2.2. Hydrolytic Stability of Thymidine 5´-Bis[3-acetyloxy-2-cyano-2-(2-phenylethylcarbamoyl)propyl] phosphate *(**1**)*

**Scheme 4:**
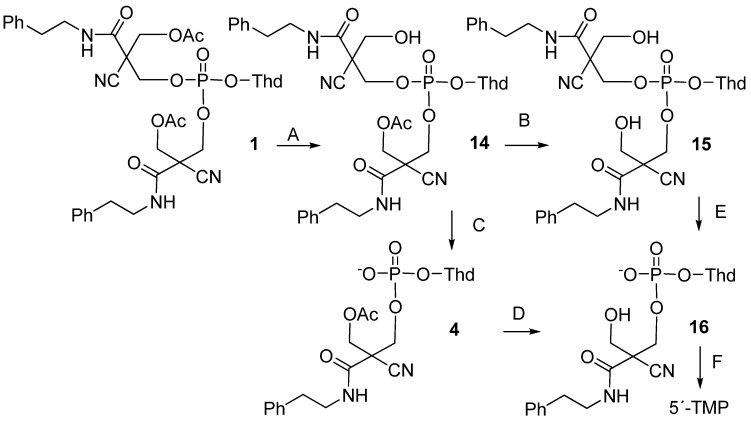
Degradation pathway of thymidine 5´-bis[3-acetyloxy-2-cyano-2-(2-phenylethylcarbamoyl)propyl]phosphate (**1**).

Hydrolysis of triester **1** was studied at pH 7.5 and 37 °C by analyzing the composition of the aliquots withdrawn from the reaction mixture at appropriate time intervals by RP HPLC. The products formed were identified by spiking with authentic samples and by mass spectrometric analysis (HPLC/ESI-MS). At pH 7.5-10, hydrolysis of the first acetic ester linkages of **1** (Reaction A in Scheme 4) was first-order in hydroxide-ion concentration. At pH 7.5, the half-life for the reaction was 28 h (*k* = 7.0 × 10^-6^ s^-1^). The subsequent departure of the remnants of this protecting group by retro-aldol condensation gave diester **4** (Route C) without accumulation of the monodeacetylated triester **14** as an intermediate. Hydrolysis of the second ester linkage (Reaction D) was 16 times slower, the half-live being 460 h. Accordingly, the compound is slightly more stable than the corresponding 3-acetyloxy-methoxy-2,2-bis(ethoxycarbonyl)propyl derived triester **2**. The half-lives for the consecutive deacetylations of **2** have been reported to be 32 h and 148 h under these conditions [29]. The 3-acetyloxy-2,2-bis(ethoxycarbonyl)propyl protected triester **3 **and its diester counterpart **6** are considerably more stable, the half-lives for the disappearance of **3** and **6** at pH 7.5 and 25 °C being 480 h and 3850 h, respectively [29].

**Figure 1:**
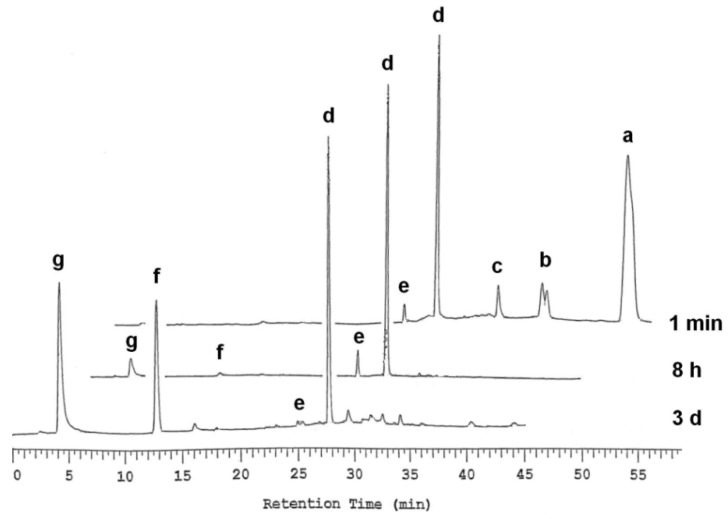
RP-HPLC profiles for the HLE-catalyzed hydrolysis of thymidine 5´-bis[3-acetyloxy-2-cyano-2-(2-phenylethylcarbamoyl)propyl]phosphate (**1**) at pH 7.5 and 37.0 °C (*I* = 0.1 mol L^-1^ with NaCl). Notation: (a) **1**, (b) **14**, (c) **15**, (d) **4**, (e) **16**, (f) thymidine and (g) 5´-TMP. For detailed chromatographic conditions, see the experimental section.

### 2.3. Enzymatic Deprotection of Thymidine 5´-Bis[3-acetyloxy-2-cyano-2-(2-phenylethylcarbamoyl)-propyl]phosphate *(**1**)*

Treatment of triester **1** with hog liver carboxyesterase (26.0 units mL^-1^) in a 2-[4-(2-hydroxyethyl)piperazin-1-yl)]ethanesulfonic acid (HEPES) buffer (3.5 mL) at pH 7.5 and 37 °C (*I* = 0.1 mol L^-1^ with NaCl) resulted in an intermediary accumulation of the *R*_P_- and *S*_P_-distereomers of monodeacetylated triester **14** ([M+H]^+^ = 825.7; Reaction A in Scheme 4), the half-life for the disappearance of **1 **(Reaction A) being 3.8 min. The subsequent retro-aldol condensation of **14** to diester **4** ([M+H]^+^ = 595.3; Reaction C) was, however, so fast that the amount of **14** remained less than 5% of the initial concentration of **1**. 

Deacetylation of **14** to the dideacetylated triester **15** ([M+H]^+^ = 783.8; Reaction B) competed with the retro-aldol condensation, representing approximately 10% of the total disappearance of **14**. Both, **4** and **15** were subsequently decomposed to diester **16** ([M+H]^+^ = 553.3; Reactions D and E) that was rapidly converted to 5´-TMP ([M+H]^+^ = 323.4; Reaction F). The half-life for the disappearance of **4** was 47 h. The level of accumulation of the deacetylated intermediate **16 **remained below 5%. Prolonged treatment with the esterase resulted in dephosphorylation of 5´-TMP to thymidine. Figure 1 gives as illustrative examples the chromatograms obtained at an early, intermediate and final stage of the deprotection of **1**.

Table 1 records the half-lives obtained for the various partial reactions with **1** and its 3-acetyloxy-2,2-bis(ethoxycarbonyl) and 3-acetyloxymethoxy-2,2-bis(ethoxycarbonyl) counterparts **3 ** and **2**, respectively. Although at the high HLE concentration employed the rates of Reactions B and C are comparable, Reaction C predominates at low enzyme concentrations. Comparison of the half-lives of Reaction D reveals that **1** is under such conditions converted to 5´-TMP 3 times as fast as **3**, but still one order of magnitude more slowly than **2**. The advantage of faster retro-aldol condensation of the intermediated derived from **1** is overcompensated by the slower enzymatic deacetylation of diester **4**. In other words, the desired overall acceleration of the exposure of 5´-TMP was not achieved.

**Table 1:** Half-lives for the partial reactions involved in hydrolysis of thymidine 5´-bis[3-acetyloxy-2-cyano-2-(2-phenylethylcarbamoyl)propyl]phosphate (**1**) to 5´-TMP at pH 7.5 and 37 °C (*I* = 0.1 mol L^-1^ with NaCl). For the reactions, see Scheme 4. [HLE] = 26 units mL^-1^. The corresponding half-lives reported previously [29] for thymidine 5´-bis[3-acetyloxymethoxy-2,2-bis(ethoxycarbonyl)propyl]phosphate (**2**) and thymidine 5´-bis[3-acetyloxy-2,2-bis(ethoxycarbonyl)propyl]phosphate (**3**) are included for reference.

	*t*_1/2 _/ min		
**Reaction**	**1**	**2**	**3**
A	3.8	0.17	3.2
D	2838	210	9060
B	≈ 2	0.90	31^a^
C	≈ 0.2	10.4	31^a^

^a^ 25 °C.

### 2.4. Reactions of 2-cyano-N-(2-phenyl)Ethylacrylamide with Glutathione

**Scheme 5:**
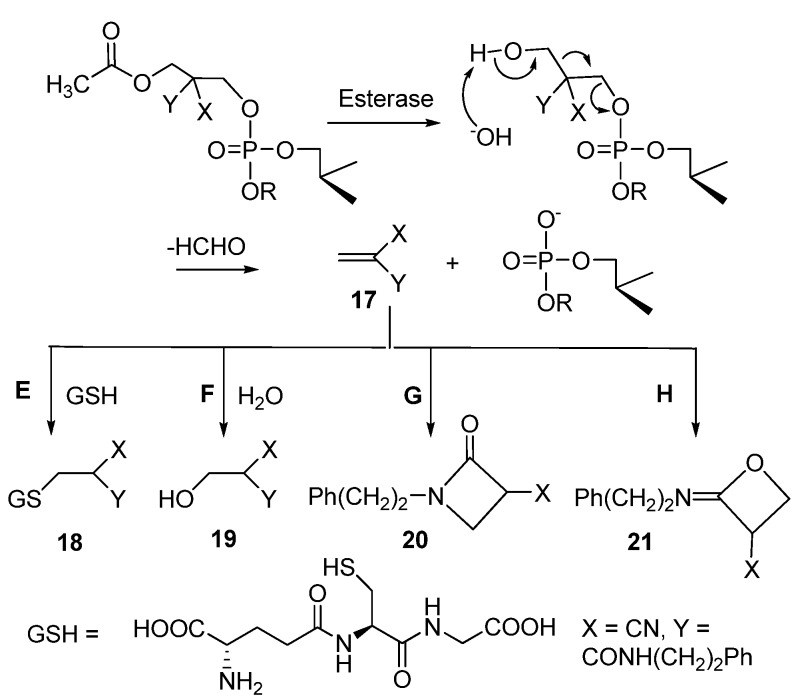
Esterase-catalyzed hydrolysis of thymidine 5´-bis[3-acetyloxy-2-cyano-2-(2-phenylethylcarbamoyl)propyl]phosphate (**1**) and reactions of 2-cyano-*N*-(2-phenyl)ethylacrylamide (**17**) by-product formed.

**Figure 2:**
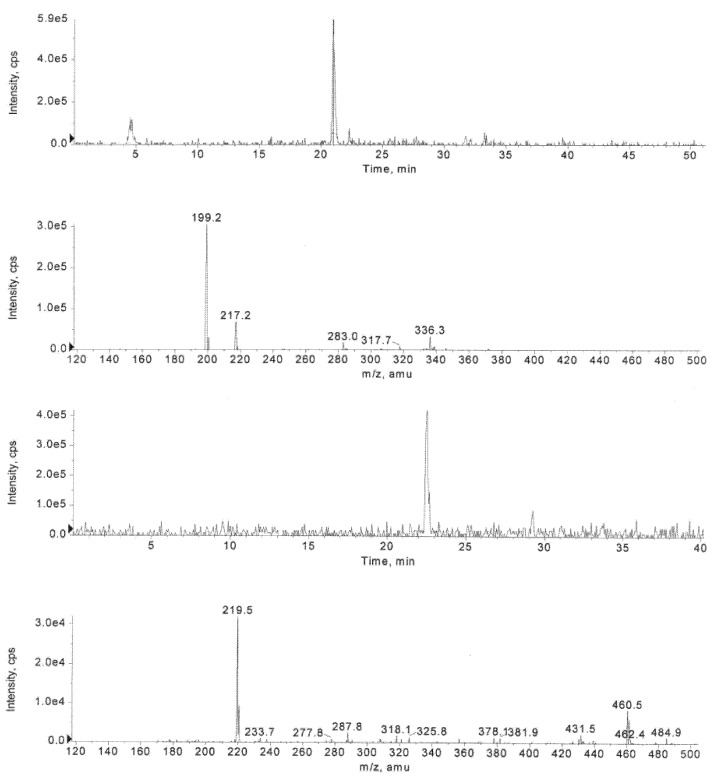
Inspection of mass spectra obtained from XIC *m/z* 217-218 (calcd. [M-H]^-^ = 217.1) and 219-220 (calcd. [M+H]^+^ = 219.1) for **19** during the hydrolysis of **1** (at *t* = 2 d) in the presence HLE at pH 7.5 and 37 °C. Chromatographic conditions: A mixture of water and MeCN containing 0.1% formic acid (linear gradient from 2% up to 60.0% MeCN in 35 min).

As discussed above, retro-aldol condensation of the deacetylated intermediates, **14**, **15** and **16**, evidently produces 2-cyano-*N*-(2-phenyl)ethylacrylamide (**17**; Scheme 5). Consistent with this suggestion, an *m*/*z* value of 199.2, referring to the molecular ion [M-H]^-^ of **17**, could be observed by the HPLC-ESI-MS analysis, but accumulation of this enone-like structure could not be verified by HPLC during the course of the hydrolysis of **1**. It should be, in addition, noted that the same molecular ion is also obtained from cyclic products **20** and **21**, formed by an intramolecular attack of either the amido oxygen (Route H) or nitrogen (Route G) on the β-carbon of **17**. In fact, the signals at *m/z* 199.2 could be observed at different elution times. The mass spectra of these compounds additionally exhibited an unidentified *m/z* peak of 187.2. The *m/z* values ([M+H]^+^ = 219.5 and [M-H]^-^ = 217.2) referring to the molecular ion of 2-cyano-3-hydroxy-*N*-(2-phenyl)ethylpropanamide **19** (see Figure 2) was visible at the late stage of hydrolysis, and, hence, hydration of **17** may take place (Route F).

**Figure 3:**
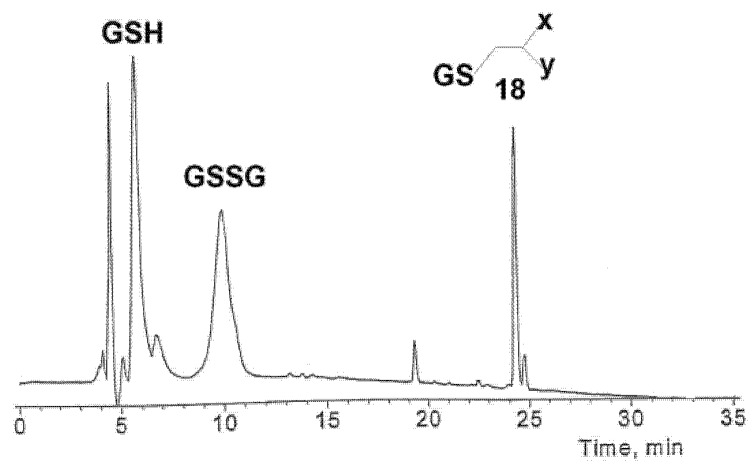
RP-HPLC profile for the glutathione conjugate of 2-cyano-*N*-phenethylacrylamide observed during the HLE-catalyzed hydrolysis of thymidine 5´-bis[3-acetyloxy-2-cyano-2-(2-phenylethylcarbamoyl)propyl]phosphate(**1**) in the presence of GSH at pH 6.2 and 37.0 °C. Chromatographic conditions: A mixture of water and MeCN containing 0.1% formic acid (linear gradient from 1% up to 50.0% MeCN in 35 min). HPLC-signals were recorded on a UV detector at a wavelength of 215 nm.

The hydrolytic reactions of **1** was also carried out in the presence of glutathione ([GSH] = 5.4 mM) and carboxyesterase ([HLE] = 28.0 units mL^-1^) in a 2-(*N*-morpholino)ethanesulfonic acid (MES) buffer (0.005/0.005 mol L^-1^) at pH 6.2 and 37 °C. Under these conditions, the half-live for the deacetylation of **1** was 18 h. In 2 days, the starting material was converted almost quantitatively to a mixture of acetylated and deacetylated phosphodiester **4** and **16** ([**4**]/[**16**] is 1:1). At this stage, 2-cyano-*N*-(2-phenyl)ethylacrylamide (**17**) was captured as the glutathione conjugate (**18**; *t*_R_ = 24.3 min, Route E). Product **18** was detected by both HPLC and LC-MS analysis ([M+H]^+^ = 508.8; see Figure 3). In addition, glutathione was converted to its oxidized form glutathione disulfide (GSSG; [M+H]^+^ = 613.5 and *t*_R_ = 9.9 min).

**Figure 4:**
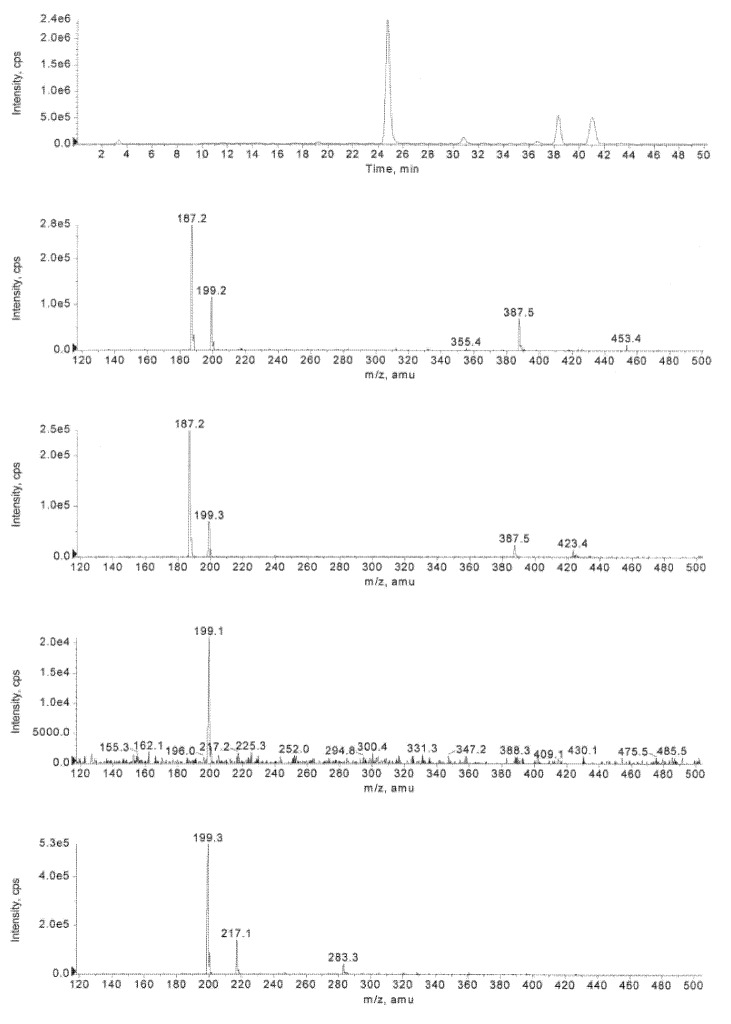
Inspection of mass spectra obtained from XIC *m/z* 217-218 (calcd. [M-H]^-^ = 217.1) and 199-200 (calcd. [M-H]^-^ = 199.1) for **19**, **20** and **21** during the hydrolysis of **1** (at *t* = 2 d) in the presence of GSH and HLE at pH 6.2 and 37 °C. Chromatographic conditions: A mixture of water and MeCN containing 0.1% formic acid (linear gradient from 1% up to 50.0% MeCN in 35 min).

Although 2-cyano-*N*-(2-phenyl)ethylacrylamide (**17**) is largely trapped by glutathione, cyclization (Routes G and H) and hydration (Route F) of **17** seem to occur as a side reaction. As seen from Figure 4, a [M-H]^-^ ion peak at *m/z* 217.3, referring most likely to 2-cyano-3-hydroxy-*N*-(2-phenyl)ethyl-propanamide (**19**), appears and undergoes fragmentation (loss of H_2_O) to yield an ion at *m/z* 199.2 (**17**). Similarly, the molecular ion signals of the cyclization products **20 **and **21** (*m/z* 199.2) were detected as in the absence of GSH. By contrast, no sign of *S*-hydroxymethylglutathione, obtained by the reaction of GSH with formaldehyde, was observed.

## 3. Experimental

### 3.1. General

Chemicals were purchased from Sigma–Aldrich, Fluka and Merck. Dichloromethane, acetonitrile, and pyridine were dried over 4Å molecular sieves. Dioxane was dried over 3Å molecular sieves. Triethylamine was dried by refluxing over CaH_2_ and distilled before use. ^1^H-, ^13^C-, ^31^P-NMR spectra were recorded on a Bruker Avance 400 (400 MHz for ^1^H, 101 MHz for ^13^C and 162 MHz for ^31^P) or 500 (500 MHz for ^1^H, 126 MHz for ^13^C and 202 MHz for ^31^P) NMR spectrometer. HRMS spectra were recorded on a Bruker Daltonics micrOTOF-Q instrument and LC-MS spectra were recorded on a Perkin-Elmer Sciex-API-365 triple-quadrupole instrument. For column chromatography, Fluka silica gel 60 (230-400 mesh) was used. Hydrolytic reactions were followed by Merck Hitachi LaChrom D7000 HPLC.

### 3.2. Materials

Hog liver carboxyesterase and glutathione were products of Fluka and Sigma, respectively. 3´-*O*-Levulinoylthymidine (**11**) and 2-cyano-3-hydroxy-2-(hydroxymethyl)-*N*-(2-phenylethyl)propanamide (**8**) were prepared as described previously [33,37]. Protected thymidine 5´-monophosphates **1** was prepared by stepwise alcoholysis of bis(diethylamino)phosphorochloridite with 3´-*O*-levulinoylthymidine (**11**) and 2-cyano-2-(hydroxymethyl)-3-oxo-3-(phenethylamino)propyl acetate (**10**), a method that has previously been used for synthesis of **2** and **3** [29].

*3´-O-Levulinoylthymidine* (**11**) was prepared as described previously [31]. ^1^H-NMR (500 MHz, CDCl_3_): *δ* = 9.36 (s broad, 1H, NH), 7.73 (s, 1H, H6), 6.25 (dd, 1H, *J* = 6.5, 2.0 Hz, H1´), 5.37 (m, 1H, *J* = 2.5 Hz, H3´), 4.11 (d, 1H, *J* = 2.0 Hz, H4´), 3.90 (m, 2H, H5´ and H5´´), 2.80 (dt, 2H, *J* = 6.0, 1.5 Hz, CH_2_ Lev), 2.59 (t, 2H, *J* = 6.0 Hz, CH_2_ Lev), 2.42 (m, 2H, H2´ and H2´´), 2.22 (s, 3H, CH_3_), 1.92 (s, 3H, CH_3_ Lev). ^13^C-NMR (126 MHz, CDCl_3_): *δ* = 206.79 (C=O Lev), 172.61 (C=O Lev), 164.11 (C4), 150.59 (C2), 136.59 (C6), 111.32 (C5), 86.04 (C1´), 85.08 (C4´), 74.96 (C3´), 62.47 (C5´), 37.83 (C2´), 37.15 (CH_2_Lev), 29.81 (CH_2_Lev), 27.95 (CH_3_ Lev), 12.55 (CH_3_). ESI^+^-MS: *m/z*obsd. 341.6 [M + H]^+^, calcd. 341.1.

*2-cyano-3-hydroxy-2-(hydroxymethyl)-N-(2-phenylethyl)propanamide* (**8**) was obtained as described previously [37]. ^1^H-NMR (400 MHz, DMSO): *δ* = 7.98 (t, 1H, *J* = 5.6 Hz, NH), 7.27 (m, 2H, Ph), 7.20 (m, 3H, Ph), 5.53 (t, 2H, *J* = 5.6 Hz, 2 × OH), 3.70-3.63 (m, 4H, *J* = 5.6 Hz, 2 × CH_2_O), 3.30 (m, 2H, CH_2_N), 2.73 (t, 2H, C*H*_2_Ph). ^13^C NMR (101 MHz, DMSO): *δ* = 165.79 (C=O), 139.73 (Ph), 129.15 (Ph), 128.79 (Ph), 126.58 (Ph), 120.15 (CN), 62.19 (CH_2_O), 55.73 (C2), 41.52 (CH_2_N), 35.36 (*C*H_2_Ph) ppm. ESI^+^-MS: *m/z* [M + Na]^+^ obsd. 271.2, calcd. 271.2.

*2-cyano-2-(hydroxymethyl)-3-oxo-3-(2-phenethylamino)propyl acetate* (**10**). Concentrated H_2_SO_4_(8.4 μL, 0.15 mmol) was added to a mixture of compound **8 **(1.54 g, 6.2 mmol) and triethyl orthoacetate (1.71 mL, 9.3 mmol) in dry THF (10 mL). The reaction was allowed to proceed overnight and the mixture was the poured into an ice-cold solution of 5% NaHCO_3_ (50 mL). The product was extracted with diethyl ether (3 × 50 mL), washed with saturated aqueous NaCl (3 × 50 mL) and dried over Na_2_SO_4_. The solvent was evaporated and the crude product, 5-cyano-2-ethoxy-2-methyl-*N*-(2-phenyl)-ethyl-1,3-dioxane-5-carboxamide (**9**), was subjected to silica gel chromatography, eluting with a mixture of DCM and MeOH (90:10, *v/v*). The product (**9**) was obtained as oil (1.5 g, 76%). ^1^H-NMR (400 MHz, DMSO) *δ* = 8.55, 8.44, 8.26, 7.98 (t each, 1H, *J* = 5.5 Hz, NH), 7.28 (m, 2H, Ph), 7.21 (m, 3H, Ph), 4.32, 4.02, 3.57, 3.45 (q each, 2H, *J* = 7 Hz, C*H_2_*CH_3_), 5.82, 5.81, 5.54, 5.53, 4.28, 4.23, 4.07, 3.99 (d each, 4H, *J* = 5.5 Hz and *J* = 11.5 Hz, CH_2_O), 3.79-3.66 and 3.41-3.29 (m, 2H, *J* = 6 Hz, CH_2_N), 2.77, 2.74, 2.56 (t each, 2H, *J* = 7.5 Hz, C*H*_2_Ph), 2.03, 1.99, 1.42 1.36 (s each, 3H, CH_3_), 1.33, 1.17, 1.15, 1.06 (t each, 3H, *J* = 7.0 Hz, CH_2_C*H*_3_). ESI^+^-MS: *m/z* [M + Na]^+ ^obsd.341.3, calcd. 341.1.

*5-cyano-2-ethoxy-2-methyl-N-(2-phenyl)-ethyl-1,3-dioxane-5-carboxamide* (**9**) (1.3 g, 4.08 mmol) was dissolved in 80% aqueous acetic acid (50 mL) and left for 1 h at room temperature. The solution was evaporated to dryness and the residue was coevaporated three times with water. The product was purified by silica gel colum chromatogaphy eluting with a mixture of DCM and MeOH (95:5, *v/v*). The product (**10**) was obtained as solid foam (1.2 g, 90%). ^1^H-NMR (400 MHz, CDCl_3_): *δ* = 8.28 (t, 1H, *J* = 5.6 Hz, NH), 7.30 (m, 2H, Ph), 7.21 (m, 3H, Ph), 5.81 (t, 1H, *J* = 5.6 Hz, OH), 4.34 (s, 2H, C*H*_2_OAc), 3.74 (t, 2H, *J* = 5.2 Hz, C*H*_2_OH), 3.31 (2H, C*H*_2_N), 2.74 (t, 2H, *J* = 7.6 Hz, C*H*_2_Ph), 2.03 (s, 3H, C(O)CH_3_). ^13^C-NMR (101 MHz, CDCl_3_): *δ* = 170.05 (C=O Ac), 164.73 (C(O)NH), 137.93 (Ph), 128.82 (Ph), 128.75 (Ph), 126.92 (Ph), 117.60 (CN), 63.41(*C*H_2_OAc), 62.96 (CH_2_OH), 51.01(C2), 41.55 (CH_2_NH), 35.27 (*C*H_2_Ph), 20.54 (CH_3_) ppm. ESI^+^-MS: *m/z* [M + Na]^+^ obsd. 313.1146, calcd. 313.1159.

*Thymidine 5´-bis*[3-acetyloxy-2-cyano-2-(2-phenylethylcarbamoyl)propyl]*phosphate* (**1**). 3’-*O*-Levulinoylthymidine (**11**) was coevaporated three times from dry pyridine and dried on P_2_O_5_ overnight. To a solution of dried **11** (0.15 g, 0.45 mmol) in dry DCM (1.2 mL), anhydrous triethylamine (0.32 mL, 2.27 mmol) and bis(diethylamino)chlorophosphine (135 µL, 0.64 mmol) was added, and the reaction mixture was stirred for 2 h under nitrogen. The product **12** was filtered through a short silica gel column eluting with a mixture of anhydrous ethyl acetate and triethylamine in hexane (60:0.5:39.5, *v/v/v*). The solvent was removed under reduced pressure and the residue was coevaporated three times from dry MeCN to remove the traces of triethylamine.The residue (0.16 g) was dissolved in dry MeCN (0.5 mL) and compound **10** (0.44 g, 1.53 mmol) in dry MeCN (0.5 mL) and tetrazole (2.72 mmol; 6.10 mL of 0.45 mol L^-1^ solution in MeCN) were added under nitrogen. The reaction mixture was stirred for 4 h at room temperature. The phosphite ester **13** formed was oxidized with I_2_ (0.1 mol L^-1^) in a mixture of THF, H_2_O and lutidine (4:2:1, v/v/v; 7 mL) by stirring overnight at room temperature. The crude product, 3´-*O*-levulinoylthymidine 5´-bis[3-acetyloxy-2-cyano-2-(2-phenylethylcarbamoyl)propyl]phosphate (**13**) was isolated by DCM/aq. NaHSO_3_ work up, and purified on a silica gel column eluted with ethylacetate, followed by a stepwise gradient from a mixture of DCM and MeOH (98:2, *v/v*) up to 30% MeOH. The purification was repeated using ethyl acetate and a mixture of DCM and MeOH (90:10, *v/v*) as an eluent. The product (**13**) obtained as solid foam was contaminated by some unidentified impurities. The product was characterizied by HPLC-MS eluting with a mixture of water and MeCN (linear gradient from 10% up to 40.0% MeCN in 40 min) (ESI^+^-MS: m/z [M + H]^+^ obsd. 965.3 calcd. 965.7 and *t*_R_ = 29.5 min) and used without further purification. Compound **13** was dissolved in dry DCM (2.0 mL) and hydrazine acetate (0.11 mmol, 10 mg) in dry MeOH (0.20 mL) was added. After 1 h, hydrazinium acetate (0.05 mmol, 4.6 mg) in a mixture of DCM (100 μL) and MeOH (20 μL) was added. The reaction was allowed to proceed for 1.5 h and the addition of hydrazinium acetate was repeated. The reaction was quenched with acetone and the mixture was evaporated to dryness. The crude product was purified by reversed phase chromatography on a Lobar RP-18 column (37 × 440 mm, 40-63 μm), eluting with a mixture of water and acetonitrile (60:40%, *v/v*). The product was obtained as solid foam (25 mg, 43%). ^1^H-NMR (400 MHz, CDCl_3_): *δ* = 8.65 (broad, 1H, NH), 7.35-20 (m, 11H, H6 and 2 × Ph), 6.72 (m, 2H, 2 × CH_2_N*H*), 6.15 (m, 1H, H1´), 4.51-4.31 (m, 11H, H3´, H5´, H5´´ and 4 × CH_2_O), 4.00 (m, 1H, H4´),3.63 (m, 2H, *J* = 6.4 Hz, CH_2_N), 3.51 (m, 2H, *J* = 6.7 Hz, CH_2_N), 2.86 (m, 4H, *J* = 6.8 Hz, 2 × C*H*_2_Ph), 2.32 (m, 2H, H2´ and H2´´), 2.08 (s, 6H, 2 × C(O)CH_3_) 2.01 (s, 3H, CH_3_). ^13^C-NMR (101 MHz, CDCl_3_): *δ* = 169.69 (C*=*OAc), 163.58 (C4), 162.34 (C(O)NH), 150.14(C2), 137.84 (Ph), 136.5 (C6), 128.85 (Ph), 128.73 (Ph), 126.93 (Ph), 116.79 (CN), 116.35 (CN), 111.33 (C5), 85.76 (C4´), 84.14 (C1´), 67.50 (C3´), 67.08 (C5´), 62.72 (*C*H_2_OAc), 50.52(C spiro), 41.96 (CH_2_NH), 35.19 (*C*H_2_Ph), 26.86 (C2´), 20.49 (C(O) *C*H_3_), 12.45 (CH_3_) ppm. ^31^P-NMR (162 MHz, CDCl_3_): *δ* = -2,53; -2,76; -3,17; -3,35 ppm (The four phosphorus signals refer tothe presence of stereogenic center in each of the phosphate protecting groups). ESI^+^-MS: *m/z* [M + H]^+^ obsd. 867.8 calcd. 867.3. ESI^+^-MS: *m/z* [M + Na]^+^ obsd. 889.2785, calcd. 889.2780.

### 3.3. Kinetic Measurements

The reactions were carried out in sealed tubes immersed in a thermostated water bath (37.0 ± 0.1 °C). The oxonium ion concentration of the reaction solution (3.5 mL) was adjusted with glycine, 2-[4-(2-hydroxyethyl)piperazin-1-yl)]ethanesulfonic acid (HEPES) and 2-(*N*-morpholino)ethanesulfonic acid (MES) buffers. The ionic strength of the solutions was adjusted to 0.1 mol L^-1 ^with sodium chloride. The hydronium ion concentration of the buffer solutions was calculated with the aid of the known p*K*_a_ values of the buffer acid under the experimental conditions. The initial substrate concentration was ca. 0.4 mmol L^-1^. 

The enzymatic hydrolysis was carried out with Hog Liver Esterase (26 units mL^-1^) in a 2-[4-(2-hydroxyethyl)piperazin-1-yl)]ethanesulfonic acid (HEPES) buffer (0.040/0.024 mol L^-1^) at pH 7.5 and 37 °C. The samples (200 μL) withdrawn at appropriate intervals were made acidic (pH 2) with 1 mol L^-1^ aqueous hydrogen chloride to inactivate the enzyme and to quench the hydrolysis, cooled in an ice-bath and filtered with minisart RC 4 filters (0.45 μm). The composition of the samples was analyzed on an ODS Hypersil C18 column (4 × 250 mm 5 μm, flow rate 1 mL min^-1^), using a mixture of acetic acid/sodium acetate buffer (0.045/0.015 mol L^-1^) and MeCN, containing ammonium chloride (0.1 mol L^-1^). A good separation of the product mixtures of **2 **was obtained on using a 5 min isocratic elution with the buffer containing 2% MeCN, followed by a linear gradient (23 min) up to 40.0% MeCN. Signals were recorded on a UV-detector at a wavelength of 267 nm. The reaction products were identified by the mass spectra (LC/MS) using a mixture of water and acetonitrile containing a formic acid (0.1%) as an eluent (Gemini C18 column (2 × 150 mm 5 μm, flow rate 200 μL min^-1^). The first order rate constants for the non-enzymatic hydrolysis of triester **1** were obtained by applying first-order rate-law to the diminution of the concentration of the starting material. The enzymatic deacetylations obeyed first-order kinetics at the high HLE concentrations employed.

## 4. Conclusions

Thymidine 5´-phosphotriester **1** bearing two 3-acetyloxy-2-cyano-2-(alkylaminocarbonyl)propyl groups undergoes esterase triggered breakdown to 5´-TMP more readily than its 3-acetyloxy-2,2-bis-(ethoxycarbonyl)propyl counterpart, but considerably less readily than the 3-acetyloxymethoxy-2,2-bis(ethoxycarbonyl)propyl derived triester. The initial enzymatic deacetylation is followed by release of the resulting 3-hydroxy-2-cyano-2-(alkylaminocarbonyl)propyl group as 2-cyano-*N*-(2-phenyl-ethyl)acrylamide (**17**). When the reaction is carried out in large excess of glutathione, **17** is largely converted to glutathione conjugate **18**. In addition, intramolecular cyclization and hydration occur as side reactions. 
